# Integrated Quad-Scanner Strategy-Based Optical Coherence Tomography for the Whole-Directional Volumetric Imaging of a Sample

**DOI:** 10.3390/s21041305

**Published:** 2021-02-11

**Authors:** Sm Abu Saleah, Daewoon Seong, Sangyeob Han, Ruchire Eranga Wijesinghe, Naresh Kumar Ravichandran, Mansik Jeon, Jeehyun Kim

**Affiliations:** 1 School of Electronic and Electrical Engineering, College of IT Engineering, Kyungpook National University, 80, Daehak-ro, Buk-gu, Daegu 41566, Korea; abu.saleah@knu.ac.kr (S.A.S.); smc7095@knu.ac.kr (D.S.); syhan850224@knu.ac.kr (S.H.); 2 Institute of Biomedical Engineering, School of Medicine, Kyungpook National University, 80, Daehak-ro, Buk-gu, Daegu 41566, Korea; 3 Department of Materials and Mechanical Technology, Faculty of Technology, University of Sri Jayewardenepura, Pitipana, Homagama 10200, Sri Lanka; erangawijesinghe@sjp.ac.lk; 4 Center for Scientific Instrumentation, Korea Basic Science Institute, 169-148, Gwahak-ro Yuseong-gu, Daejeon 34133, Korea; nareshr9169@kbsi.re.kr

**Keywords:** optical coherence tomography, quad-scanner scanning strategy, whole-directional scanning, full-directional imaging

## Abstract

Whole-directional scanning methodology is required to observe distinctive features of an entire physical structure with a three dimensional (3D) visualization. However, the implementation of whole-directional scanning is challenging for conventional optical coherence tomography (OCT), which scans a limited portion of the sample by utilizing unidirectional and bidirectional scanning methods. Therefore, in this paper an integrated quad-scanner (QS) strategy-based OCT method was implemented to obtain the whole-directional volumetry of a sample by employing four scanning arms installed around the sample. The simultaneous and sequential image acquisition capabilities are the conceptual key points of the proposed QS-OCT method, and were implemented using four precisely aligned scanning arms and applied in a complementary way according to the experimental criteria. To assess the feasibility of obtaining whole-directional morphological structures, a roll of Scotch tape, an ex vivo mouse heart, and kidney specimens were imaged and independently obtained tissue images at different directions were delicately merged to compose the 3D volume data set. The results revealed the potential merits of QS-OCT-based whole-directional imaging, which can be a favorable inspection method for various discoveries that require the dynamic coordinates of the whole physical structure.

## 1. Introduction

Whole-directional (i.e., full-directional) scanning has been widely applied and utilized for various medical imaging techniques to identify distinctive features of samples [1,2]. Magnetic resonance imaging (MRI) and computed tomography (CT) are representative existing imaging techniques that have been actively applied for the whole-body imaging of samples [3,4]. Moreover, positron emission tomography (PET) and PET/CT techniques have also been demonstrated to obtain the whole-body imaging of samples [5]. These imaging systems are widely used for the diagnosis of cancer because whole-body imaging enables the inspection of the entire sample through a single scanning attempt. In accordance with the development of high-resolution whole-body imaging at the human level, full-body scanning methods have been developed to match the sample characteristics, such as acquiring tractography for whole mouse heart [6], brain [7], and the quantitative analysis of embryos [8]. The aforementioned imaging modalities are suitable for full-body imaging at the human level because of their deep penetration depth. However, these imaging techniques are limited by their low resolution; therefore, an optical imaging modality such as optical coherence tomography (OCT) could be a suitable solution for acquiring high-resolution morphological images of a 3D sample at the tissue level.

OCT is a non-invasive optical imaging technique used to obtain high-resolution, cross-sectional images of inner microstructures in materials and biological tissues [9]. OCT has been widely applied in various fields, including medical diagnosis [10,11], dentistry [12,13], and industrial applications [14,15]. The single-scanner-based unidirectional scanning of conventional OCT has been applied in numerous applications [16,17]. Unidirectional scanning OCT has been widely utilized for high-resolution imaging and has been utilized in various system designs, such as optical Doppler tomography [18], OCT angiography [19], and polarization-sensitive OCT [20]. However, conducting whole-directional volumetric screening is challenging with these systems because it involves the single-side imaging of a 3D sample. Therefore, the bidirectional scanning method was implemented to compensate for the limitation of single-side scanning methods by imaging dual sides of a sample, which is necessary for measuring the thickness and overlapping morphological structures of thin and high-refractive-index samples [21,22]. However, the imaging results of bidirectional scanning are affected by the sample shape and thickness, limiting its applicability for full-directional imaging.

To assess the full-directional morphological structure of a target sample, the rotational imaging (RI) strategy was demonstrated by rotating the sample stage multiple times. RI-OCT was initially implemented for in situ embryonic imaging to obtain structural information from different angles [23,24]. However, the RI-OCT system has some drawbacks, as shifting the sample direction by rotating the sample stage and adjusting the imaging focus after every rotation requires a long image acquisition time. To reduce the acquisition time, a parallel imaging scheme was introduced by scanning multiple locations of a sample concurrently. Parallel images were obtained simultaneously from multiple locations of a sample by adding optical path length delays that are longer than the light penetration depth of tissue in a parallel channel [25]. Space-division multiplexing (SDM) OCT is a representative parallel-imaging technique that achieves an improved imaging speed [26]. The SDM-OCT system was demonstrated to achieve the efficient imaging of a single-directional morphological structure using 8-beam multiplexing [27] and has been indicated for a clinical feasibility analysis of its ophthalmic applications [25].

In this study, we demonstrated a QS methodology-based OCT imaging technique to obtain whole-directional tomographic images of a sample without the rotation of the sample stage. To satisfy the different imaging criteria, such as whole-directional simultaneous imaging and unidirectional sequential imaging, we propose applying two different types of QS-OCT concepts: simultaneous and sequential modes. In simultaneous QS-OCT, the whole-directional imaging of a 3D sample was scanned from four different directions concurrently using the SDM technique of parallel imaging, which enhanced the imaging speed of the system. To verify the possibility of simultaneous QS-OCT implementation, a rolled Scotch tape sample was imaged from the whole-direction of the sample concurrently. In addition, sequential QS-OCT was demonstrated to obtain whole-directional volumetric images of a 3D sample using four scanners operated in a successive order and to address the drawbacks of the power loss of the simultaneous QS-OCT approach. To verify the capability of the whole-directional volumetric imaging of the sequential QS-OCT system, ex vivo mouse heart and kidney specimens were imaged and merged. Therefore, the QS-OCT concept in simultaneous and sequential modes is a whole-directional imaging modality that supports different imaging criteria and can obtain whole-directional morphological images without any rotation of the sample.

## 2. Materials and Methods

### 2.1. Optical Configuration and Duty Cycle Illustration of Simultaenous QS-OCT

**Figure 1:**
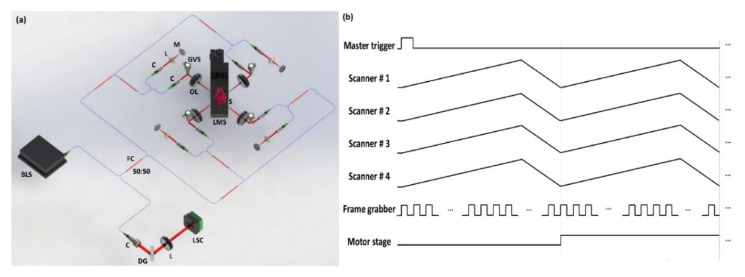
The optical configuration and duty cycle illustration of the simultaneous quad-scanner (QS)-OCT system. (**a**) Simultaneous QS-OCT with space-division multiplexing for simultaneous whole-directional imaging. (**b**) The duty cycles of simultaneous QS-OCT system operation. BLS, broadband light source; C, collimator; DG, diffraction grating; FC, fiber coupler; GVS, galvanometer scanner; L, lens; LMS, linear motor stage; LSC, line-scan camera; OL, objective lens; S, sample.

The schematic of the optical configuration for simultaneous QS-OCT is shown in Figure 1a. The system was equipped with a broadband light source (EXS210090-01, Exalos, Zurich, Swiss) with a central wavelength of 840 nm, a full width at half maximum bandwidth of 48 nm, and an average output power of 15 mW. Four one-axis galvanometer-scanners (GVS001, Thorlabs, Newton, NJ, USA) were mounted at four sides of a sample stage to cover the full-directional scanning of the sample. In each sample arm, a 2-inch object lens (AC508-100-B, Thorlabs, Newton, NJ, USA) was used for large-area scanning. Four reference arms, identically designed with a collimator (F260APC-B, Thorlabs, Newton, NJ, USA), lens (AC254-030-B, Thorlabs, Newton, NJ, USA), and mirror (PF10-03-P01, Thorlabs, Newton, NJ, USA), were used for the four sample arms in this system. The power of each interferometer was equally divided and maintained at the saturation level of the detector. The ratio of all fiber couplers (TW850R5A2, Thorlabs, Newton, NJ, USA) utilized in this system was 50:50. Polarization controllers (FPC023, Thorlabs, Newton, NJ, USA) were utilized in each reference arm and sample arm to regulate the polarized state of the transmitted light. To apply the SDM technique for obtaining whole-directional images concurrently, the optical path length of each interferometer was accurately controlled. Each interference signal obtained by four different interferometers was transferred to a customized spectrometer, whose configuration was described in detail in [28]. A frame grabber (PCIe-1433, National Instruments, Austin, TX, USA) and data acquisition board (DAQ, PCIe-6323, National Instruments, Austin, TX, USA) were employed to precisely control the hardware compositions. A linear motor stage (M-403, PI, Karlsruhe, Germany) was used to move the sample stage up and down to acquire the 3D volumetric imaging of the sample.

The synchronized operation of different hardware instruments (four scanners, frame grabber, and linear motor stage) is essential to precisely obtain whole-directional morphological images of a sample at the same time. Therefore, the duty cycle illustration explains the sequence of operation, which was employed in simultaneous QS-OCT system setups, as shown in Figure 1b. In accordance with the rising edge of the main trigger from DAQ, four scanners and a frame grabber were synchronized concurrently for scanning and grabbing. Although four sample arms were implemented independently, each scanner was controlled with identical operating timing by sawtooth waves. Moreover, the frame grabber was precisely controlled according to the grabbing timing, which was determined by the utilized A-scan rate of the scanner. In the proposed QS-OCT (both simultaneous and sequential), the operations of the scanners started with 20 kHz A-line rates following the rising edge of the main trigger signal. In addition, the frame grabber and motor stage were started simultaneously, with intervals for grabbing and moving identically set as 50 μs to be matched with the A-scan rate. After obtaining single B-scan images, the motor stage was moved immediately as much as the preset step size for measuring the whole volume of the sample. The aforementioned scanning process was continued for the total range of the sample and the whole-directional volumetric raw data were obtained using the proposed simultaneous QS-OCT system.

### 2.2. Optical Configuration and Duty Cycles of Sequential QS-OCT

**Figure 2:**
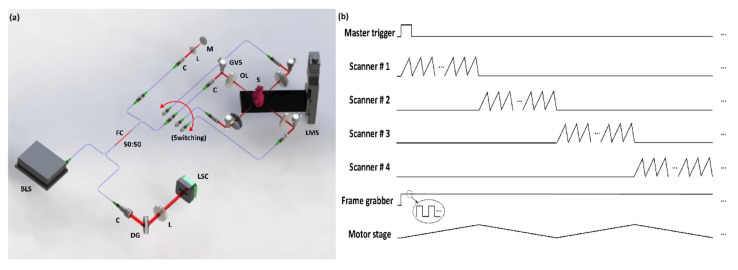
The optical configuration and duty cycle diagram of sequential quad-scanner (QS)-OCT system. (**a**) Sequential QS-OCT for successive operation of each scanner. (**b**) The duty cycle illustration of sequential QS-OCT system operation. BLS, broadband light source; C, collimator; DG, diffraction grating; FC, fiber coupler; GVS, galvanometer scanner; L, lens; LMS, linear motor stage; LSC, line-scan camera; OL, objective lens; S, sample.

Figure 2a shows the schematic of the optical configuration of the sequential QS-OCT for whole-directional volumetric imaging that resolves the power limitations of the simultaneous method. In the case of a simultaneous strategy, SDM was implemented for parallel imaging to obtain whole-directional images simultaneously. Although the simultaneous system offered a fast scanning speed, the power of the detected interference signal was comparably low, leading to the degradation of image intensity at high frequencies (depth direction in the imaging window), because four different scanners were each equipped with one interferometer and the source power was divided into four interferometers. In contrast, as a feature of the sequential QS-OCT system, each scanner was operated successively in a systematically fixed order to improve the transferred power of each sample arm. The optical components used in the sequential system (including source, fiber coupler, sample arm, reference arm, and spectrometer) were the same as those used in the simultaneous system. Unlike the simultaneous method, one common reference arm was used for four sample arms in the sequential QS-OCT system to reduce the number of fiber couplers used in the simultaneous QS-OCT system. A motorized linear stage, utilized in the simultaneous method as well, was implemented for scanning the whole-directional volumetric imaging where the switching between individual scanners was performed manually.

Likewise, a synchronization process for operating the simultaneous system and the hardware compositions of sequential QS-OCT method were controlled according to precisely synchronized timing, as shown in Figure 2b. The overall operating timing is identical to that of the simultaneous method; however, the scanners operated independently according to the switching sequence to enhance the power of the system. After acquiring a single B-scan image, the motor stage was moved to the next point according to the timing of a single B-scan acquisition, but the direction of movement was conversely changed for efficient scanning (i.e., upward (#1 and #3) and downward (#2 and #4)). As a result of whole-direction imaging by following the precisely controlled operating order, 3D volumetric raw data were obtained and processed by customized merging software.

### 2.3. The Description and the Flow Chart of the Image Processing Algorithm

**Figure 3:**
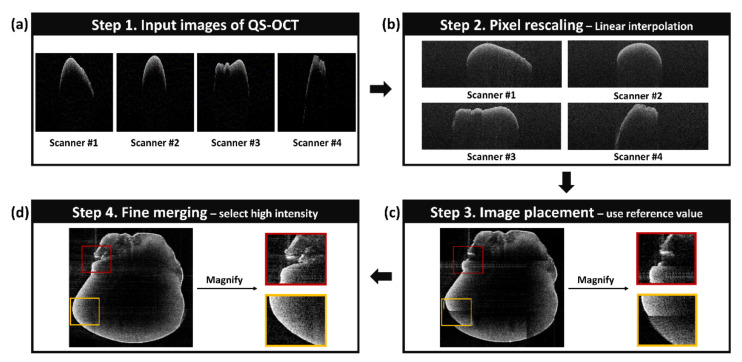
The description and flow chart of the image processing algorithm for obtaining the whole-directional volumetric data of the sample. (**a**–**d**) demonstrate each step of customized algorithm for merging cross-sectional OCT images obtained from quad-scanner methods.

To obtain a whole-directional volumetric data set, we developed a LabVIEW (National Instruments, Austin, TX, USA) -based customized merging software and the operational flowchart of this software (described in Figure 3). The first step of the software was used to classify the images acquired from four different scanners using simultaneous and sequential methods, respectively. In the case of the simultaneous method, the input image separation process was required, since the obtained B-scan image was composed with different signals of 4 independent scanners by employing SDM for the enhancement of imaging speed. Separated images matched with each scanner were obtained by replacing the value to zero, excepting the signals of each region-of-interest. In contrast, the separation process for the input image is not required in sequential QS-OCT, which scans four different directions successively. Four different images obtained from each direction acquired by both simultaneous and sequential QS-OCT—as shown in Figure 3a—were transferred to the post-processing part, including pixel rescaling, image placement, and fine merging. Pixel rescaling, the second step of the customized algorithm, was applied for adjusting the pixel resolution difference between the lateral (14.7 μm) and axial (4.69 μm) direction. The difference in pixel resolution between the lateral and axial direction affected the accuracy of the merging process, which requires a comparison of every pixel’s position. To match the pixel resolution, linear interpolation was applied to the lateral direction according to the ratio difference between the lateral and axial direction. Next, interpolated images, which are shown in Figure 3b, were placed at each direction to conduct the image merging process. Prior to the imaging of QS-OCT, a 3D-printed cylindrical sample, which had features around the surface to indicate the overlapped position, was imaged to obtain a reference value of image merging. Therefore, as shown in Figure 3c, interpolated images of each scanner were placed in accordance with the pre-acquired reference value of pixel movement. In addition, to finely merge the whole-directional images, we compared intensities and selected higher values for the overlapped regions, based on the fact that the intensity of OCT signal, measured at the focal point, is higher than at other positions. To compare the quality of merged images between step 3 (image placement) and step 4 (fine merging), two representative regions (red and yellow squares) were selected and magnified. The result of applying the fine merging method, as shown in Figure 3d, demonstrates a distinctive outer and inner structure and smoothly connected edge lines, proving the validity of selecting a higher intensity for overlapped regions. By utilizing the demonstrated customized merging software, we obtained whole-directional volumetric images of the sample shown in the results section.

### 2.4. Collection of Mouse Heart and Kidney Specimens

The mouse heart and kidney specimens utilized in this study were extracted from a 6-week-old BALB/c mouse and were harvested immediately after sacrificing the mouse. The extracted heart and kidney were preserved in 10% neutral-buffered formalin at room temperature for five days. All the animal experimental procedures were proceeded in conformity with a laboratory animal protocol approved by the Institutional Animal and Human Care and Use Committee of Kyungpook National University (No. KNU-2020-0025).

## 3. Results

### 3.1. Quantitative Analysis of Performance and Alignment of Scanners

**Figure 4:**
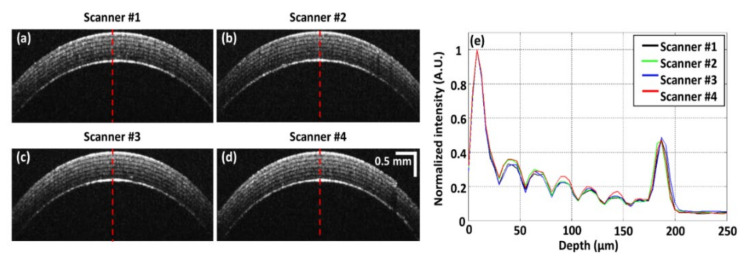
Quantitative performance assessment of each sample arm in quad-scanner (QS)-OCT; (**a**–**d**) are B-scan images of a Scotch tape roll obtained by sequentially switching the scanner; (**e**) demonstrates the A-scan profiling results of images (**a**–**d**) centered on the red-dashed line to quantitatively analyze performance.

To quantitatively analyze the performance and alignment of each scanner, which are crucial factors for obtaining whole-directional volumetric imaging, a rolled Scotch tape sample was scanned using four scanners from four directions, the acquired cross-sectional OCT images of which are shown in Figure 4a–d. While analyzing the scanner performance, the power of each scanner was equally controlled to objectively compare the image quality. All seven layers of the Scotch tape roll were distinguished in each cross-sectional image (Figure 4a–d) that was obtained using sequential QS-OCT. Moreover, to quantitatively assess the performance of each scanner, A-scan profiling was conducted, as shown in Figure 4e, and was acquired from the middle position of the Scotch tape cross-sectional images, indicated by the red dashed line in Figure 4a–d. The black, green, blue, and red profiles in Figure 4e represent the A-scans of each scanner from #1 to #4, respectively. The acquired A-scan result of the rolled Scotch tape revealed distinguishable internal layers along with information about the peak intensity, with an approximately similar peak height. According to the A-scan profiling results, the quality of each image acquired from different directions using QS-OCT was identical and reliable for conducting whole-directional imaging and merging to create a 3D volumetric image.

**Figure 5:**
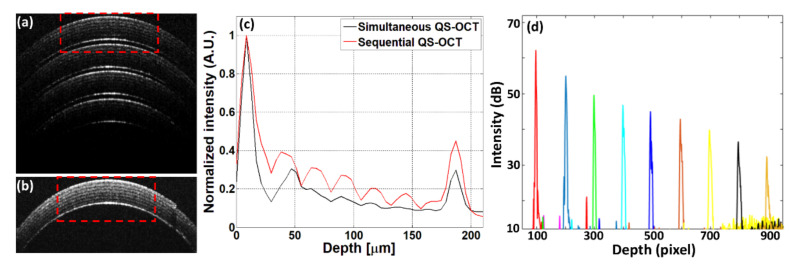
The performance difference between the simultaneous and sequential quad-scanner (QS) OCT system and the measured intensity fall-off of the proposed system. (**a**) is the cross-sectional OCT image of the simultaneous QS-OCT system. (**b**) is the cross-sectional OCT image of the sequential QS-OCT system. (**c**) is the depth intensity profiles of the simultaneous and sequential QS-OCT systems. (**d**) is the measured intensity fall-off graph of the proposed system obtained every 100 pixels.

The performance difference between simultaneous and sequential QS-OCT and the measured intensity fall-off graph are shown in Figure 5. Figure 5a,b show the cross-sectional OCT images of rolled scotch tape which were obtained from the simultaneous and sequential QS-OCT system, respectively. The red dotted boxes in Figure 5a,b indicate the ROIs, from where the depth intensity profiles were measured. Figure 5c shows the depth intensity profiles of both systems. A total of 150 A-lines were taken from the ROI, and then these A-lines were averaged to form the depth intensity profiles of the simultaneous and sequential QS-OCT system, where it is visualized that the depth intensity profile of sequential QS-OCT is higher than the depth intensity profile of simultaneous QS-OCT. Though internal layers of the Scotch tape were distinguished in both simultaneous and sequential QS-OCT, the overall intensities of each layer were found to be higher in the sequential method. In addition, as a case of sensitivity roll-off of the proposed system, the backscattered intensity was measured at every 100 pixels (from 100th to 900th) using a mirror as a target sample. As shown in Figure 5d, 28 dB was dropped at a depth of 4.1 mm compared to the top surface, which was caused by the characteristic of SD-OCT. Moreover, the lateral resolution was measured as 15.6 μm utilizing a resolution target (USAF 1951, Edmund Optics, Barrington, NJ, USA), and the axial resolution was calculated as 6.47 μm in air.

### 3.2. Imaging Process and Measured Data Using Simultaneous QS-OCT

**Figure 6:**
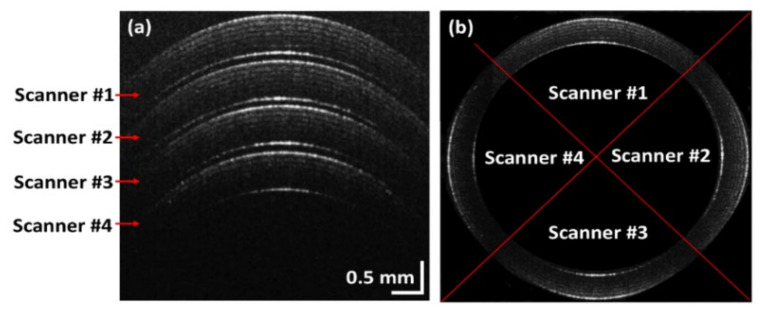
Simultaneously obtained images of a Scotch tape roll with the space-division multiplexing technique applied using a simultaneous quad-scanner (QS)-OCT. (**a**) is a representative B-scan image of the rolled Scotch tape imaged from four different directions with simultaneous QS-OCT, (**b**) is a merged image of the four QS-OCT images shown in (**a**).

To explain the functional process of simultaneous QS-OCT, cross-section and merged OCT images are shown in Figure 6, obtained from the rolled Scotch tape sample used in Figure 4. The cross-sectional image of the Scotch tape roll, as shown in Figure 6a, was obtained simultaneously from whole-directions using the SDM technique with simultaneous QS-OCT. The Scotch tape cross-sectional images acquired from four scanners are indicated by red arrows. Because the difference between the path length of the sample and reference arm gradually increased towards the bottom of the imaging window, the measured intensity reduced according to the depth direction. Figure 6b exhibits the merged cross-sectional OCT images of the rolled Scotch tape that were shown in Figure 6a. The cross-sectional OCT images of the rolled Scotch tape sample shown in Figure 6a,b indicate the parallel imaging capability of simultaneous QS-OCT with the SDM imaging technique, as well as the drawback of power loss in the depth direction of the imaging window, leading to the degradation of image intensity. Although the intensity of the rolled Scotch tape image decreased in the depth direction due to the imaging depth limitation of SD-OCT, the possibility of using the proposed simultaneous QS-OCT method to concurrently obtain whole-directional images was demonstrated and is one of the proposed proofs-of-concept for whole-directional imaging.

### 3.3. The Examination of Merged 3D Data of Ex Vivo Mouse Kideny and Heart Obtained by Sequential

**Figure 7:**
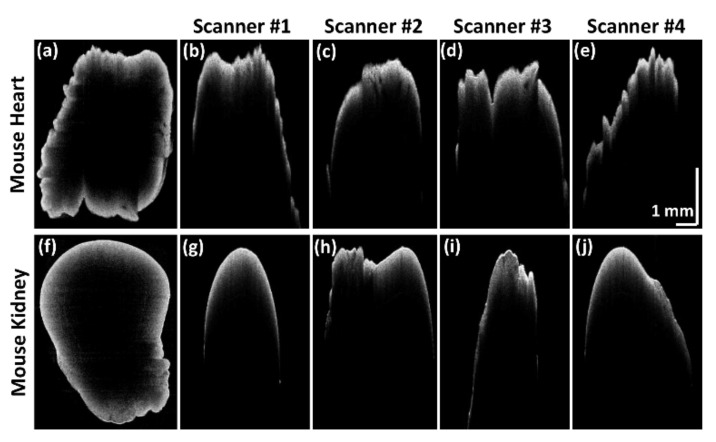
Enface and cross-sectional OCT images of mouse heart and kidney specimens. (**a**) and (**f**) are the enface images of mouse heart and kidney, respectively. (**b**–**e**), and (**g**–**j**) are the cross-sectional OCT images of mouse heart and kidney, respectively.

Sequential QS-OCT was developed to overcome the aforementioned limitation of power loss that occurs in the simultaneous QS-OCT system. Sequential QS-OCT utilizes a common reference arm for four different scanners to compensate for the power loss. To verify the performance of sequential QS-OCT in assessing the whole-directional imaging of biological samples, we obtained whole-directional morphological images of a mouse heart and kidney as ex vivo specimens. Following the set value of the A-scan rate (20 kHz), the volumetric imaging of each scanner consumed 36.25 s with 0.0125 μm step sizes of motor movement. Figure 7 shows the cross-sectional OCT images and their merged enface images of mouse heart and kidney specimens. The cross-sectional OCT images shown in Figure 7b–e and Figure 7g–j were obtained using four scanners through whole-directional imaging. These four cross-sectional OCT images were merged using an image processing algorithm, described in Figure 3, to form the enface images shown in Figure 7a,f. All the characteristic features that are seen in the cross-sectional OCT images of the mouse heart and kidney specimens are retained in their respective enface images.

**Figure 8:**
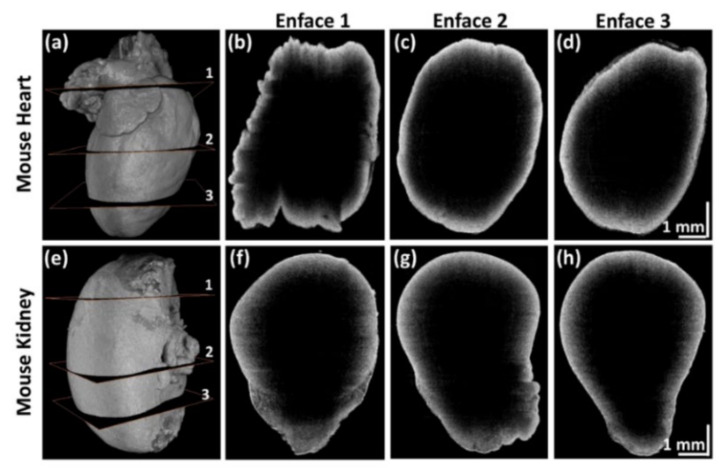
3D volumetric and representative enface images of mouse heart and kidney using quad-scanner (QS)-OCT. (**a**,**e**) are full-directional 3D rendering morphological images of a mouse heart and kidney; (**b**–**d**) and (**f**–**h**) are the selected enface images obtained at the three different layers shown in (**a**,**e**), respectively.

Figure 8 shows the 3D volumetric and enface images of mouse heart and kidney specimens acquired in sequential QS-OCT. Full-directional 3D rendering morphological images of the mouse heart and kidney are shown in Figure 8a,e, respectively. Every side of the sample was scanned vertically using a linear motor stage for volumetric imaging (1000 × 2048 × 725 pixels). Independently obtained volumetric images from four different sample arms were merged to obtain enface images with customized rendering software. Figure 8b–d,f–h are the representative enface images that were obtained from three different layers of 3D volumetric imaging of mouse heart and kidney specimens, shown in Figure 8a,e, respectively. The enface images shown in Figure 8b–d,f–h demonstrate the proper merging process for obtaining whole-directional volumetric data of the sample. The obtained 3D volumetric and enface images of the mouse heart and kidney specimens verify the whole-directional imaging capability of QS-OCT.

## 4. Discussion

In this study, the whole-directional volumetric imaging strategy for full-directional morphological assessment of a 3D sample without additional sample rotation was successfully demonstrated in QS-OCT, where the assessment was challenged in conventional unidirectional and bidirectional scanning-based OCT. Conventional unidirectional scanning OCT has mostly been used for acquiring single-side cross-sectional images of a sample [16,17]. In addition, bidirectional imaging, which is based on the simultaneous top and bottom surface scanning of the sample, is limited by the sample thickness and outer shape [21]. In contrast, the proposed QS-OCT methods (simultaneous and sequential modes) scan from the whole-directions of the sample, which makes dynamic assessment possible for the entire position of the sample. Rotational imaging is a whole-directional imaging method in which additional sample handling such as the rotation of the sample stage and the adjustment of focus after every rotation are essential to continue the imaging capture. In the case of QS-OCT, however, the path length of each scanner was accurately controlled throughout the entire imaging process.

In simultaneous QS-OCT, whole-directional images were acquired simultaneously to enhance the imaging speed by applying the SDM technique. However, the division of source power into four reference arms and four sample arms was the major drawback of the simultaneous QS-OCT system, leading to degradation of the image intensity at high frequencies. Averaged irradiation power to sample of each scanner, utilizing simultaneous and sequential method, were measured as 6.79% and 36.4% compared with the source, which verified the significant decrement of power in simultaneous method. The drawback of source power division into multiple reference and sample arms can be compensated using a high-power light source. The sequential QS-OCT imaging concept has been demonstrated to overcome the power loss caused by the simultaneous method. In sequential QS-OCT, a common reference arm was used for the four sample arms to maintain the high power needed to conduct whole-directional imaging. The mutual compensation characteristics of simultaneous and sequential methods of the proposed QS-OCT concept can be utilized based on application requirements for scanning speed and division of source power. In the case of simultaneous QS-OCT, this method could be a suitable solution for the dynamic whole-directional volumetric assessment of a sample using a fast scanning speed. In contrast, sequential QS-OCT could be a suitable technique for obtaining the whole-directional volumetric imaging of a sample with an enhanced image quality.

As an aspect of SDM applied to simultaneous QS-OCT, the degradation of the image intensity in depth direction, as shown in Figure 6, was caused by the short coherence length of SD-OCT. In addition, the SD-OCT based quad-scanner with SDM has the restraint of applications, which is proper to utilize for thin samples because of the limited number of camera pixels. The limitation of a short coherence length while using SD-OCT can be compensated by using swept-source OCT, which has a comparatively longer coherence length to capture multiple images in the imaging depth [29]. However, the proposed simultaneous QS-OCT method was applied to obtain the volumetric imaging of a sample using the SDM technique with four scanners, whereas the SDM technique was conventionally used using a single scanner in other applications [26].

## 5. Conclusions

The proposed methodology of whole-directional scanning serves as a proof of concept for the whole-directional volumetric imaging of a 3D sample using QS-OCT in simultaneous or sequential order. Simultaneous QS-OCT was developed with the SDM technique by mounting four precisely aligned sample arms around the sample stage to achieve whole-directional volumetric imaging with a fast imaging speed. A sequential QS-OCT concept has also been demonstrated to improve image quality while using fewer fiber couplers than the simultaneous QS-OCT system by compromising the imaging speed. The performance of the scanners was quantitatively analyzed by imaging the Scotch tape with an A-scan profiling result. The applicability of the proposed simultaneous and sequential QS-OCT concepts was demonstrated with merged and volumetric images of the Scotch tape roll and biological tissues (heart and kidney of a mouse), respectively. In conclusion, the possibility of QS-OCT-based whole-directional imaging was achieved, and the proposed methods could be useful in various fields that require a dynamic assessment of the entire position of a sample, such as material testing and optical inspection, to detect product defects.

## Data Availability

Not applicable.
